# Goal-directed intraoperative therapy based on autocalibrated arterial pressure waveform analysis reduces hospital stay in high-risk surgical patients: a randomized, controlled trial

**DOI:** 10.1186/cc8875

**Published:** 2010-02-15

**Authors:** Jochen Mayer, Joachim Boldt, Andinet M Mengistu, Kerstin D Röhm, Stefan Suttner

**Affiliations:** 1 Department of Anesthesiology and Intensive Care Medicine, Klinikum Ludwigshafen, Bremserstrasse, 79, 67063 Ludwigshafen, Germany

## Abstract

**Introduction:**

Several studies have shown that goal-directed hemodynamic and fluid optimization may result in improved outcome. However, the methods used were either invasive or had other limitations. The aim of this study was to perform intraoperative goal-directed therapy with a minimally invasive, easy to use device (FloTrac/Vigileo), and to evaluate possible improvements in patient outcome determined by the duration of hospital stay and the incidence of complications compared to a standard management protocol.

**Methods:**

In this randomized, controlled trial 60 high-risk patients scheduled for major abdominal surgery were included. Patients were allocated into either an enhanced hemodynamic monitoring group using a cardiac index based intraoperative optimization protocol (FloTrac/Vigileo device, GDT-group, n = 30) or a standard management group (Control-group, n = 30), based on standard monitoring data.

**Results:**

The median duration of hospital stay was significantly reduced in the GDT-group with 15 (12 - 17.75) days versus 19 (14 - 23.5) days (*P *= 0.006) and fewer patients developed complications than in the Control-group [6 patients (20%) versus 15 patients (50%), *P *= 0.03]. The total number of complications was reduced in the GDT-group (17 versus 49 complications, *P *= 0.001).

**Conclusions:**

In high-risk patients undergoing major abdominal surgery, implementation of an intraoperative goal-directed hemodynamic optimization protocol using the FloTrac/Vigileo device was associated with a reduced length of hospital stay and a lower incidence of complications compared to a standard management protocol.

**Trial Registration:**

**Clinical trial registration information: **Unique identifier: NCT00549419

## Introduction

There is growing evidence that perioperative goal-directed therapy (GDT) based on flow-related hemodynamic parameters improves patient outcome [1,2], particularly in high-risk patients [3,4]. Mean arterial blood pressure (MAP) and central venous pressure (CVP) are routinely used to monitor hemodynamics, but no information on blood flow can be obtained with MAP and CVP. Therefore, enhanced hemodynamic monitoring seems to be crucial in the guidance of perioperative volume therapy and cardiocirculatory support. Previous optimization studies vary largely with regard to study design and the complexity of the monitoring technique used. Most of the trials used the pulmonary artery catheter (PAC) [5-8] and the esophagus Doppler (ED) method [9-11]. These methods are either highly invasive (PAC) or show limited accuracy (ED) [12] combined with other disadvantages such as frequent dislocation of the ultrasound probe [13] or poor toleration in awake patients [14].

In the present study, we used the FloTrac/Vigileo, a minimally invasive device, which only needs standard arterial access for enhanced, flow-based hemodynamic monitoring. The device is reported to be easy to use and easy to set up [15] and calculates the stroke volume on the basis of the arterial waveform in combination with demographic data. Recent studies have shown a good agreement compared with more invasive methods to determine cardiac output (CO) [16-19]. In this study we aimed to determine whether an intraoperative optimization protocol using the enhanced flow-based hemodynamic parameters of the FloTrac/Vigileo device would result in an improvement in outcome in high-risk patients undergoing major abdominal surgery, measured by the length of hospital stay (LOS) compared with a standard protocol based on conventional hemodynamic data.

## Materials and methods

After obtaining written informed consent and Institutional Review Board approval, 60 patients with an American Society of Anesthesiologists (ASA) physical status (Table 1) [20] of III with two or more risk factors according to risk index of Lee (Table 2) [21] undergoing open major abdominal surgery (intestine resection, gastric resection, liver resection, esophageal resection, Whipple) were studied between 18 January 2008 and 16 March 2009. Patients under 18 years, patients with severe aortic regurgitation, permanent cardiac arrhythmias, intra-aortic balloon pump and patients undergoing emergency surgery were excluded from the study.

**Table 1:** The American Society of Anesthesiologists (ASA) physical status

ASA physical status	Description
I	A normal healthy patient
II	A patient with mild systemic disease
III	A patient with severe systemic disease
IV	A patient with severe systemic disease that is a constant threat to life
V	A moribund patient who is not expected to survive without the operation

**Table 2:** The revised Lee cardiac risk index

1. High-risk type of surgery
2. Ischemic heart disease
3. History of congestive heart failure
4. History of cerebrovascular disease
5. Insulin therapy for diabetes
6. Preoperative serum creatinine > 2.0 mg/dl

The study was a single-centre, prospective randomized trial carried out in a tertiary, university affiliated hospital. Patients were randomized preoperatively either into a standard protocol group (control group) or an enhanced, goal-directed hemodynamic monitoring group (GDT group) using a closed envelope system. Randomization was performed by a member of the research team.

In both groups, premedication consisted of midazolam (0.01 mg kg^-1^), and standard general anesthesia was induced with fentanyl 1 to 2 μg kg^-1^, propofol 1.5 to 2 mg kg^-1 ^and cisatracrurium 0.07 mg kg^-1^. After intubation of the trachea, the lungs were ventilated to maintain normocapnia (end expiratory partial pressure of carbon dioxide level 32 to 38 mmHg) using a constant fresh gas flow of 1 L min^-1^. Maintenance of anesthesia was performed with 0.9 to 1.8% end tidal sevoflurane, and fentanyl and cisatracrurium boli were given as needed. Standard monitoring for both groups included electrocardiogram, invasive arterial blood pressure via right or left radial artery, CVP, pulse oximetry, temperature, inspiratory and expiratory gas concentrations.

**Figure 1:**
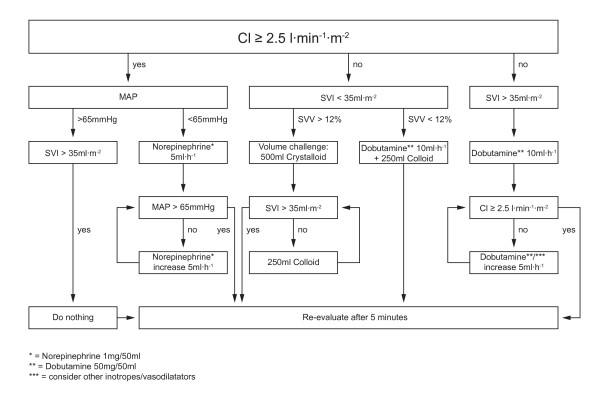
**Enhanced hemodynamic monitoring protocol with FloTrac/Vigileo**. CI, cardiac index; MAP, mean arterial pressure; SVI, stroke volume index; SVV, stroke volume variation.

**Figure 2:**
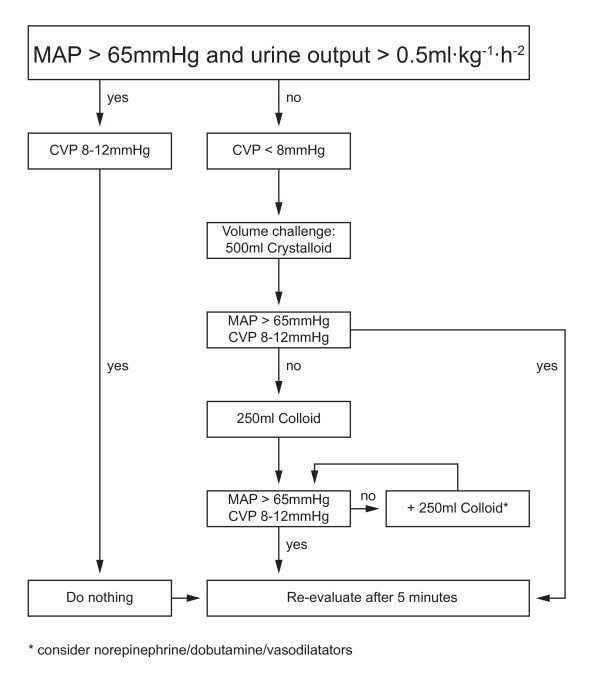
**Standard care protocol**. MAP, mean arterial pressure; CVP, central venous pressure.

In the control group, MAP was kept between 65 and 90 mmHg, CVP between 8 and 12 mmHg and urinary output more than 0.5 mL kg^-1 ^h^-1^. The GDT-group patients received enhanced hemodynamic monitoring with the FloTrac/Vigileo device (Edwards Lifesciences, Irvine, CA, USA) and an attempted cardiac index (CI) of at least 2.5 L·min^-1^·m^-2^. The arterial line was connected to the Vigileo monitor (software version 1.14; Edwards Lifesciences, Irvine, CA, USA) via the FloTrac pressure transducer and all intravascular pressure measurements were referenced to mid-axillary line level. The shape of the arterial curve was checked visually for damping throughout the study period. CI, stroke volume index (SVI), as an indicator for fluid status, and stroke volume variation, (SVV) as an indicator for fluid responsiveness during mechanical ventilation and sinus rhythm, were continuously measured. Details of the protocols for both standard and enhanced hemodynamic monitoring are summarized in Figures 1 and 2. Side effects of GDT (e.g. tachycardia during dobutamine infusion) were not acceptable and as soon as they developed further optimization attempts were ceased and patients were kept at the best possible level. Blood loss was substituted with fluids according to the protocols and a hemoglobin value below 8 mg dL^-1 ^was considered to be a trigger for transfusion of packed red blood cells.

The respective protocols in both groups were continued until the transportation monitoring equipment was attached to the patients, which happened after the end of surgery and hemodynamic stability. All patients were admitted to the intensive care unit (ICU) and both groups were managed by the same physicians on the same wards (ICU and general ward) who were not involved in the intraoperative management, data collection or group allocation of the study. Complications were assessed daily by senior anesthesiologists and senior surgeons blinded to group allocation and study design using standard predefined criteria. All data were collected by a study nurse blinded to the study design and group allocation, except vital data, which were collected automatically using custom PC software (NarkoData, Imeso, Hüttenberg, Germany).

To ascertain comparable preconditions between the groups with respect to preoperative co-morbidity and type of surgery, all patients underwent POSSUM (physiological and operative severity score for the enumeration of mortality and morbidity) scoring [22].

Patients were ready for hospital discharge when they showed stable cardiovascular and respiratory conditions, ability to take oral fluids, sufficient pain control, mobilization (as far as possible), spontaneous micturition, infection parameters within normal range, consciousness comparable with the preoperative state and non-irritated wound conditions. These criteria were classified by specialist surgeons, who where not involved in the study design or group allocation.

### Statistical analysis

The primary outcome variable was the duration of hospital stay. Secondary outcome variables were the incidence of perioperative complications, the duration of the ICU stay, the amount and type of fluids used intraoperatively, and the amount and type of vasoactive and positive inotropic support used intraoperatively.

A MedCalc 4.31 software package (MedCalc Software, Mariakerke, Belgium) was used for statistical analyses. The number of patients required in each group was determined before the study by a power calculation based on the results of a similar previous study [1]. It was found that the minimum clinically important difference we wished to detect was a 20% decrease in the primary endpoint duration of hospital stay. With an assumed α error of 0.05 (two-sided) and type II error of 0.2, we found 24 patients per group to be required. To compensate for possible dropouts, we decided to include 30 patients per group.

The assumption of normality was checked using the Kolmogorov-Smirnov test. Continuous, normally distributed data were compared using paired and unpaired Student's t-test and a Bonferroni correction for repeated measurements was applied. Continuous, non-normally distributed data were compared using the Wilcoxon test. Binominal data were compared using chi-squared analysis and Fisher's exact test. All tests were two-sided and were performed at a corrected α = 0.05 level unless otherwise specified.

## Results

**Figure 3:**
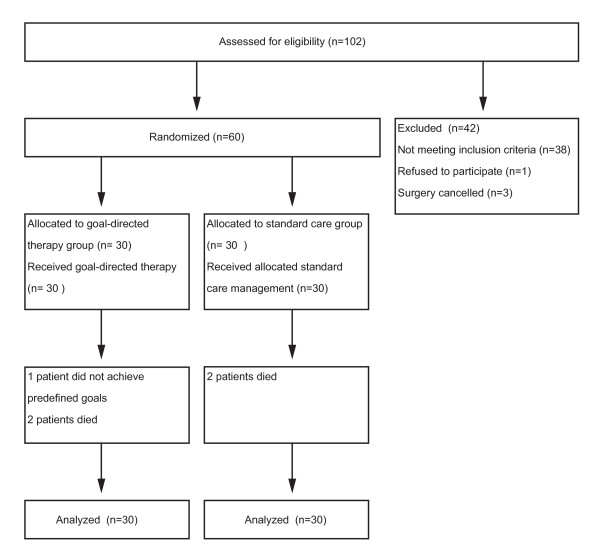
**Patient flow throughout the study**.

**Figure 4:**
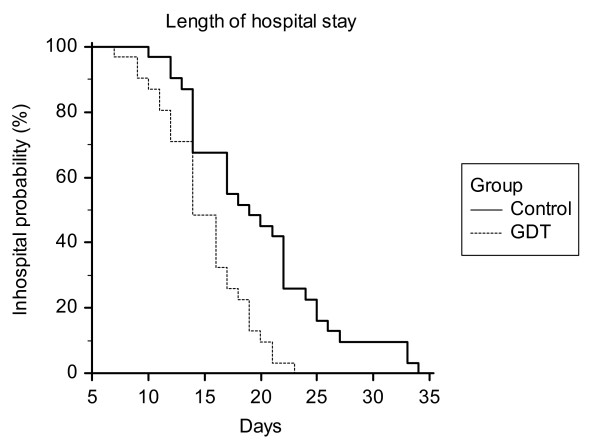
**Kaplan-Meier survival analysis of length of hospital stay**. The dotted line represents the goal-directed therapy (GDT) group.

The patient flow through the study is shown in Figure 3. Both groups were comparable with respect to age, gender, weight, co-morbidities and the type of surgery as determined by the Lee classification scheme (Table 2) [21] and the POSSUM score [22] (Table 3). Anesthetic requirements and duration of surgery also did not differ between the groups (Table 4). In the GDT group, we found a reduced median (interquartile range) duration of hospital stay of 15 (12 to 17.75) days versus 19 (14 to 23.5; *P *= 0.006; Figure 4) in the control group. The number of patients who developed complications was lower in the GDT group (6 patients, 20%) than in the control group (15 patients, 50%; *P *= 0.03) and fewer complications per group were documented in the GDT group (17 complications) than in the control group (49 complications; *P *= 0.001; Table 5). No difference was found between the groups in the duration of ICU stay (39.6 ± 39.5 hours in the GDT group vs. 41.9 ± 43.5 hours in the control group; *P *= 0.70) and postoperative mechanical ventilation (4.8 ± 4.5 hours in the GDT group vs. 7.8 ± 10.0 hours in the control group; *P *= 0.14). Significantly more colloids were administered in the GDT group (1188 ± 550 ml vs. 817 ± 467 ml; *P *= 0.006), whereas the amount of crystalloid volume replacement was lower (2489 ± 805 ml vs. 3153 ± 1264 ml; *P *= 0.02). The total amount of fluids administered intraoperatively (including packed red blood cells and fresh frozen plasma) was not different between the groups (4528 ± 2317 ml vs. 4494 ± 1561 ml). Positive inotropic support with dobutamine was higher in the GDT group (30.4 ± 50.5 μg kg^-1 ^h^-2 ^vs. 4.1 ± 19.0 μg kg^-1^h^-2^; *P *= 0.01). Administration of norepinephrine, epinephrine and nitrates was similar between the groups. No difference was found with regard to urinary output, loss of blood and blood transfusion. One patient in the GDT group did not achieve the predefined goals and optimization attempts were ceased because of tachyarrhythmia with a CI around 2.2 L·min^-1^·m^-2^. All patients of the control group achieved the predefined goals. Two postoperative deaths occurred in each group. In each group, one patient died secondary to anastomotic leakage and sepsis. In one patient of the control group, myocardial infarction was diagnosed leading to fatal cardiogenic shock. One patient of the intervention group developed massive intraabdominal bleeding, which was fatal before emergency re-laparotomy could be performed.

**Table 3:** Demographic and preoperative data

	GDT group n = 30	Control group n = 30
Gender (m/f)	20/10	22/8
Age (years)	73 (69-78)	72 (68-78)
Body mass Index (kg·m^-2^)	25.8 ± 3.8	26.4 ± 5.5
POSSUM score		
Physiology	22 (19-25)	21 (19-27)
Operation	17 (15-22)	19 (15-21)
Surgical procedure		
Hemicolectomy	10	11
Gastrectomy	10	5
Rectum resection	3	9
Whipple	5	2
Esophagus resection	1	1
Liver resection	1	2
Pre-existing conditions		
Ischemic heart disease	20	18
Cerebrovascular disease	6	5
Diabetes mellitus requiring insulin	2	2
Hypertension	27	28
Obstructive pulmonary disease	3	4
Peripheral vascular disease	4	4
Renal failure requiring dialysis	0	0
Renal failure without dialysis	6	5

All data presented as mean ± standard deviation, except age and POSSUM score values (median (interquartile range)). GDT, goal-directed therapy; f, female; m, male; POSSUM, physiological and operative severity score for the enumeration of mortality and morbidity [22].

**Table 4:** Intraoperative data, hemodynamics and volume replacement

	GDT group n = 30	Control group n = 30	*P*
Duration of anesthesia (min)	357 ± 92	365 ± 113	0.75
Surgery time (min)	280 ± 84	297 ± 109	0.51
Urinary output (ml·kg^-1^·h^-1^)	2.2 ± 1.5	1.6 ± 1.6	0.16
Blood loss (ml)	1090 ± 1385	892 ± 747	0.49
Intraoperative hemodynamics^#^			
Heart rate (bpm)	69 ± 15	70 ± 16	0.31
MAP (mmHg)	80.6 ± 16.1	74.6 ± 15.5	0.006*
CVP (mmHg)	12 ± 5	10 ± 4	0.01*
SVI (ml m^-2^)	38.8 ± 9.1	-	-
SVRI (dyne·s·cm^-5^·m^-2^)	2101 ± 459	-	-
CI (L·min^-1^·m^-2^)	2.7 ± 0.8	-	*-*
Crystalloid volume replacement (ml)	2489 ± 805	3153 ± 1264	0.02*
Colloid volume replacement (ml)	1188 ± 550	817 ± 467	0.006*
PRBC (ml·kg^-1^·h^-2^)	1.3 ± 1.8	0.9 ± 1.0	0.28
FFP (ml·kg^-1^·h^-2^)	0.5 ± 1.3	0.2 ± 1.6	0.35
Total volume infused intraoperatively (ml)	4528 ± 2317	4494 ± 1561	0.95

#, mean of values taken automatically every five minutes; * significant; bpm, beats per minute; CI, cardiac index; CVP, central venous pressure; GDT, goal-directed therapy; FFP, fresh frozen plasma; MAP, mean arterial pressure; PRBC, packed red blood cells; SVI, stroke volume index; SVRI, systemic vascular resistance index. All data presented as mean ± standard deviation.

**Table 5:** Complications until hospital discharge

Complication	Diagnostic tools	GDT group n = 30	Control group n = 30
**Infection**			
Pneumonia	Confirmed chest x-ray, WBC > 12 × 10^3 ^or < 4 × 10^3 ^ml^-1^	1	3
Abdominal	Abdominal CT	1	4
Urinary tract	Dysuria, urine analysis	0	0
Wound	Clinical diagnosis	3	8
**Respiratory**			
Pulmonary embolism	CTPA	0	0
Respiratory support > 24 hours or weaning failure	NIV > 24 hours, Re-intubation	2	3
**Cardiovascular**			
Pulmonary edema	Auscultation, chest x-ray	0	2
Arrhythmia	≥ Lown II, ≥ 30 atrial extrasystoles, AF, VF	2	3
Hypotension	Mean arterial pressure ≤ 50 mmHg	2	9
Acute myocardial infarction	ECG signs for ischemia, troponin T ≥ 0.03 ng ml^-1^	0	2
Stroke	Clinical diagnosis confirmed with CCT	0	1
**Abdominal**			
Bowel obstruction	No defecation > 4 days	1	2
Upper gastro-intestinal bleeding	Clinical diagnosis, confirmed with endoscopy	1	0
Anastomotic leak	Drainage discharge, abdominal CT, WBC > 12 × 10^3 ^or < 4 × 10^3 ^ml^-1^	1	3
**Renal**			
Urine output < 500 ml/day or required dialysis for acute renal failure	Clinical diagnosis	1	5
**Post-operative massive hemorrhage**	> 300 ml h^-1 ^and/or need of re-operation	0	2
**Perioperative deaths**		2	2
**Total number of complications**		17	49
**Number (percentage) of patients with complications**		6 (20%)	15 (50%)

AF, atrial fibrillation; CCT, cranial computed tomography;CT, computed tomography; CTPA, computed tomography pulmonary angiogram; ECG, electrocardiogram; GDT, goal-directed therapy; NIV, non invasive ventilation; VF, ventricular flutter; WBC, white blood cell count.

Two patients in each group were actually discharged two days later than possible because of social reasons.

## Discussion

Intraoperative GDT based on minimally invasive, flow-related parameters obtained by autocalibrated arterial waveform analysis resulted in a significant reduction in LOS and significantly less perioperative complications compared with a standard management protocol with pressure-based target parameters.

The first evidence that flow-based cardiovascular parameters such as CO or oxygen delivery index (DO_2_I) correlate with the outcome in high-risk patients or high-risk surgery was shown by Shoemaker and colleagues [23,24]. Although these studies remained controversial, subsequent work confirmed that goal-directed protocols for perioperative management using flow-related parameters improve patient outcome [1-3,5-8,25,26]. The underlying mechanisms of the success of GDT are not yet entirely clear. Most authors assume that an oxygen debt from decreased blood flow, hypoxia or hypovolemia may cause mitochondrial damage and subsequent organ dysfunction [27]. Thus, adequate tissue oxygen supply seems to play a key role to prevent adverse patient outcome. Although blood flow to peripheral tissues is difficult to measure, tissue oxygen supply may be approximated using the DO_2_I. However, the DO_2_I needs to be calculated from information provided by repeated blood gas analyses. We therefore decided to use the CI as the target variable of the GDT protocol in this study, because this variable can be easily obtained and continuously measured with the arterial waveform analysis method in a busy intraoperative setting. Together with adequate hemoglobin levels and arterial oxygen saturation, we considered the CI as an adequate target for flow-based GDT.

The results of this study are in good agreement with previous trials dealing with goal-directed hemodynamic optimization based on flow-related parameters, although target variables and methods to achieve the goals vary widely in the literature. Lithium indicator dilution was used by Pearse and colleagues [1] to determine CO and DO_2_I in patients undergoing major abdominal surgery. In this study, patients in the intervention group were optimized postoperatively with colloids and dopexamine to achieve a DO_2_I of 600 ml min^-1 ^m^-2^. A significant reduction in LOS from 29.5 days to 17.5 days and in the number of patients with complications (69% vs. 44%) were found in comparison to a CVP-based protocol in a standard care group. POSSUM score values and surgical interventions were comparable with the present study, but Pearse and colleagues initiated their optimization protocol later with admission to ICU. The Lithium dilution cardiac output (LiDCO) method was used, which is considered more invasive and complicated than autocalibrated arterial waveform analysis because frequent manual recalibrations are required [28] and an artificial indicator limits the number of calibrations per day [29]. Lopes and colleagues [25] analyzed the effects of intraoperative optimization of pulse pressure variation (PPV). PPV was kept below 10% with colloid boluses in the intervention group and a significant reduction in LOS (from 17 to 7 days) and complications (75% of the patients vs. 41% of the patients) was found. In contrast to the present study, no protocol for the control group existed and PPV was the only parameter to guide optimization. Several previous studies used ED as the GDT, but were mostly limited to fluid optimization [19,10,11]. Noblett and colleagues [11] investigated the effects of ED-guided intraoperative colloid fluid resuscitation in patients undergoing colorectal resection and found a reduced LOS (nine vs. six days) and a reduced complication rate. The median POSSUM scores, however, were lower in this study (explaining the shorter LOS), administration of inotropes was not part of the optimization protocol and no protocol for the standard care group existed. The role of the ED method in goal-directed fluid therapy was investigated in a meta-analysis by Abbas and Hill [26] and an overall reduction of LOS and lower complication rates were found in the GDT groups of five studies, although absolute CO measurements were found to be imprecise [12].

In the present study, the amount of colloids administered in the GDT group was significantly higher and the amount of crystalloids was lower, which could have been protocol dependant. However, this finding is consistent with findings in other GDT literature, where a trend towards a more generous administration of colloids instead of crystalloids can be seen [1,2,25,30] and may be most likely a result of an earlier detection of fluid demand with enhanced hemodynamic monitoring. Kimberger and colleagues [31] recently investigated the influence of different volume regimens on tissue perfusion in an animal model and found a significantly increased microcirculatory blood flow and tissue oxygen tension with goal-directed administration of colloids. The ongoing discussion about the 'optimal' amount and type of fluid can at least partially be resolved, as evidence grows that individually titrated, goal-directed administration of primarily colloid solutions improves patient outcome in patients undergoing major abdominal surgery [2,25,32].

Permanent cardiac arrhythmias are a problem that affects almost all methods to determine flow-based hemodynamic variables, in particular those using the arterial waveform as source of information. The precision becomes less accurate and determination of SVV is not possible. Although temporary, short arrhythmic episodes can be eliminated by the algorithm of the Vigileo device, episodes shorter than five minutes were eliminated by ceasing measurements during this time. We also had to exclude patients with permanent cardiac arrhythmias, which might be a limitation of this study. It has also been found that the bolus administration of vasoactive drugs may affect accuracy of the arterial waveform-based method [33]. However, bolus administration was rarely necessary and measurements were discontinued during this period. Furthermore, the study is underpowered to analyze mortality and patient follow-up was performed until hospital discharge only.

## Conclusions

The results of this study demonstrated that an optimization protocol based on flow-related hemodynamic parameters obtained with the minimally invasive FloTrac/Vigileo device reduced the duration of hospital stay and perioperative complications in high-risk patients undergoing major abdominal surgery.

## Key messages

• Intraoperative GDT using a protocol based on enhanced hemodynamic variables derived by the FloTrac/Vigileo device reduced the LOS in high-risk patients undergoing major abdominal surgery compared with a standard management protocol.

• The incidence of complications was reduced in the enhanced monitoring group.

• No difference between the standard and enhanced monitoring protocol groups was found with regard to ICU stay.

## Abbreviations

ASA: American Society of Anesthesiology; CI: cardiac index; CO: cardiac output; CVP: central venous pressure; DO_2_I: oxygen delivery index; ED: esophagus Doppler; GDT: goal-directed therapy; ICU: intensive care unit; LiDCO: lithium dilution cardiac output; LOS: length of hospital stay; MAP: mean arterial pressure; PAC: pulmonary artery catheter; POSSUM: physiological and operative severity score for the enumeration of mortality and morbidity; PPV: pulse pressure variation; SVI: stroke volume index; SVV: stroke volume variation.

## Competing interests

JM and JB received speaking fees from Edwards Lifesciences, Irvine, CA, USA.

## Authors' contributions

JM and SS conceived and designed the study, performed the statistical data analysis and drafted the manuscript. JM and JB were responsible for patient recruitment. AM and KR participated in data acquisition. All authors read and approved the final manuscript.
